# Oncotype DX breast cancer recurrence score can be predicted with a novel nomogram using clinicopathologic data

**DOI:** 10.1007/s10549-017-4170-3

**Published:** 2017-02-27

**Authors:** Amila Orucevic, John L. Bell, Alison P. McNabb, Robert E. Heidel

**Affiliations:** 1 Department of Pathology, University of Tennessee Medical Center, 1924 Alcoa Hwy, Knoxville, TN 37920 USA; 2 Department of Surgery, University of Tennessee Medical Center, 1924 Alcoa Hwy, Knoxville, TN 37920 USA; 3 Graduate School of Medicine, University of Tennessee Medical Center, 1924 Alcoa Hwy, Knoxville, TN 37920 USA

**Keywords:** Oncotype DX breast cancer recurrence score, Nomograms predicting Oncotype DX results, Clinicopathologic variables predict Oncotype DX score

## Abstract

**Purpose:**

Oncotype DX (ODX) recurrence score (RS) breast cancer (BC) assay is costly, and performed in only ~1/3 of estrogen receptor (ER)-positive BC patients in the USA. We have now developed a user-friendly nomogram surrogate prediction model for ODX based on a large dataset from the National Cancer Data Base (NCDB) to assist in selecting patients for which further ODX testing may not be necessary and as a surrogate for patients for which ODX testing is not affordable or available.

**Methods:**

Six clinicopathologic variables of 27,719 ODX-tested ER+/HER2−/lymph node-negative patients with 6–50 mm tumor size captured by the NCDB from 2010 to 2012 were assessed with logistic regression to predict high-risk or low-risk ODXRS test results with TAILORx-trial and commercial cut-off values; 12,763 ODX-tested patients in 2013 were used for external validation. The predictive accuracy of the regression model was yielded using a Receiver Operator Characteristic analysis. Model fit was analyzed by plotting the predicted probabilities against the actual probabilities. A user-friendly calculator version of nomograms is available online at the University of Tennessee Medical Center website (Knoxville, TN).

**Results:**

Grade and progesterone receptor status were the highest predictors of both low-risk and high-risk ODXRS, followed by age, tumor size, histologic tumor type and lymph-vascular invasion (C-indexes-.0.85 vs. 0.88 for TAILORx-trial vs. commercial cut-off values, respectively).

**Conclusion:**

**T**his is the first study of this scale showing confidently that clinicopathologic variables can be used for prediction of low-risk or high-risk ODXRS using our nomogram models. These novel nomograms will be useful tools to help physicians and patients decide whether further ODX testing is necessary and are excellent surrogates for patients for which ODX testing is not affordable or available.

**Electronic supplementary material:**

The online version of this article (doi:10.1007/s10549-017-4170-3) contains supplementary material, which is available to authorized users.

## Introduction

Oncotype DX (ODX) (Genomic Health, Redwood City, CA) is a commercially available 21-gene breast cancer recurrence score assay, which has both prognostic and predictive value for estrogen receptor-positive (ER+)/human epidermal growth factor receptor 2-negative (HER2-)/lymph node-negative breast cancer patients [1]. The ODX recurrence score predicts benefit of adding adjuvant chemotherapy to hormonal manipulation [2]. ODX testing is currently endorsed by American Society of Clinical Oncology (ASCO), the National Comprehensive Cancer Network (NCCN), and others for routine guideline application [3–5].

ODX is costly, a factor which contributes to the test being performed in only approximately one-third of eligible breast cancer patients in the United States [6]. It is estimated that ODX is used in less than 20% of patients in European countries due to limited access and reimbursement [7]. Disparities of its use in the United States have recently been published [6, 8–10].

A few recent institution-based studies attempted to predict the results of ODX test using a limited number of respective institutions’ patients tested with ODX [11–15]. They correlated the results with certain histopathologic variables available from pathology reports such as immunohistochemical expressions of ER and progesterone receptor (PR), immunohistochemical expression or fluorescence in situ hybridization results for HER2, as well as tumor grade, and tumor size [11–15]. A majority of the models also used immunohistochemical expression of Ki-67 proliferation index in spite of its controversy in routine testing of breast cancers [16].

The objective of our study is to develop user-friendly nomograms to be used as surrogate prediction models for a high-risk or a low-risk ODX recurrence score test results. The nomogram development is based on the large breast cancer dataset from the National Cancer Data Base (NCDB), using six common and readily available clinicopathologic variables established in clinical practice as prognostic and/or predictive. These variables are: age, tumor size, tumor grade, PR status, lymph-vascular invasion (LVI), and histologic type of breast cancer (four most common types).

## Materials and methods

### Patients and pathology variables selection

The methods published by Iasonos et al. [17] were used to construct nomograms to predict for a high-risk or a low-risk ODX recurrence score based on the commercial cut-off recurrence score values (0–17 = low-risk and 31–100 = high-risk) or Trial Assigning IndiviuaLized Options for Treatment (NCT00310180)-TAILORx clinical trial (TAILORx-trial) cut-off values (0–10 = low-risk and 26–100 = high-risk). Breast cancer patients tested with ODX assay from 2010 to 2012 and with results recorded as a numerical value (0–100) were identified in the NCDB and served as the study cohort.

The NCDB, a clinical oncology database, acquires data from cancer registries from more than 1,500 Commission on Cancer-accredited facilities with estimated capture of 70% of all newly diagnosed malignancies in the United States [18]. The NCDB de-identified data regarding the names of patients and institutions prior to the release of the data files. Since criteria of 45 CFR 46.102 d research were met, Institutional Review Board approval was not required.

Inclusion criteria for creation of the nomograms were: (1) female, (2) invasive breast carcinoma, (3) ER positive, (4) HER2 negative, (5) no regional lymph node metastasis, and (6) tumor size between 6 mm and 50 mm. Our inclusion criteria are the same as the ones recommended as eligibility criteria for ODX testing by the newest 2016 ASCO clinical practice guidelines [3]. Patients also were required to have one of the four most frequent histologic types of breast carcinoma: invasive ductal, invasive lobular, invasive ductal and lobular, or invasive ductal carcinoma mixed with other types. Patients with intermediate ODX score results were excluded, since guidelines for the role of adjuvant chemotherapy in this group of patients remains under investigation in an ongoing prospective TAILORx clinical trial.

The outcome of interest for the nomograms was the probability of a high-risk or a low-risk ODX recurrence score.

### Nomogram development and statistical methods

Simultaneous logistic regression models were used to construct the nomograms. Tumor size and age were considered continuous variables, tumor grade and histology were ordinal, and LVI and PR were coded as follows: 0 = absent LVI, 0 = PR-negative; 1 = present LVI, 1 = PR-positive. The outcome variable was also similarly coded with 0 = low-risk ODX recurrence score as a reference category and 1 = high-risk ODX recurrence score as the outcome of interest when predicting for a high-risk ODX recurrence score. Coded outcome variables were reversed when predicting for a low-risk ODX recurrence score. Multicollinearity among the predictor variables was assessed using the variance inflation factor (VIF). VIF values at or above 2.5 assumed evidence of multicollinearity in a model. Calibration of the model was checked by plotting the predicted probabilities against the actual probabilities. Discrimination of the model was assessed using a receiver operating characteristic (ROC) curve to yield a concordance index (c-index) with 95% confidence interval (95% CI).

In order to validate the original model, a second independent sample of patients was collected from the NCDB from breast cancer patients tested with ODX assay in 2013, and with results recorded as a numerical value (0–100). The logistic regression model was performed with the same predictor and outcome variables. Multicollinearity, calibration, and discrimination of the validation model were assessed using the same methods. The findings of the original model and the validation model were then compared.

In order to create a nomogram from the predicted probabilities, a scoring system from 0 to 100 was created. To calculate scores based on possessing predictor characteristics, the beta coefficients (*β*) for each predictor variable were given numerical point values. The predictor variable with the highest *β* was assigned 100 points. Then, each remaining β was ranked, divided by the highest *β*, and multiplied by 100 to yield their respective point values. For age and tumor size, absolute maximum β values were assigned by multiplying the raw *β* value by the range for age or tumor size, respectively. The point values for each variable in the model were summed and linked to their respective probabilities of having a high-risk or a low-risk ODX recurrence score. Larger values denoted a higher probability of having a high-risk or a low-risk ODX recurrence score. All statistical analyses were conducted using SPSS Version 22 (Armonk, NY: IBM Corp.), and then confirmed in R program (Vienna, Austria) [19–21].

An easy, user-friendly online nomogram calculator was developed in order to expedite the calculations of the probability of a low-risk or a high-risk ODX score for each patient and is available at the University of Tennessee Medical Center web site: https://gsm.utmck.edu/nomograms.

## Results

The original cohort of patients captured by the NCDB between 2010 and 2012 consisted of 27,685 ODX-tested patients when applying commercial cut-off values for a low-risk or a high-risk recurrence score. The external validation cohort consisted of 12,763 ODX-tested patients captured by the NCDB in 2013. Descriptive characteristics of these patients with six clinicopathologic variables chosen for a nomogram creation are contrasted in Tables 1 and 2. Age, tumor size, and PR were found to be statistically significantly different when the original cohort was compared to the external validation cohort of patients (<.05). However, these difference were not considered clinically significant.

**Table 1:** Frequencies of a low-risk and a high-risk OncotypeDX (ODX) recurrence score with commercial cut-off values and pathologic descriptive characteristics

Characteristics	No	Original cohort NCDB 2010-2012 Commercial cut-off values		No	Validation cohort NCDB 2013 Commercial cut-off values		*p* value
No of patients		ODX Low risk (0–17) No (% total)	ODX High risk (31–100) No (% total)		ODX Low risk (0–17) No (% total)	ODX High risk (31–100) No (% total)	
Sex—female	27,685	23,937 (86.5)	3748 (13.5)	12763	11,185 (87.6)	1578 (12.4)	
Age (20–90) (mean ± SD)	27,685 58.43 ± 10.3	23,937 (86.5)	3748 (13.5)	12763 59.04 ± 10.3	11,185 (87.6)	1578 (12.4)	<.001
≤40	1055	837 (3.0)	218 (0.8)	440	338 (2.6)	102 (0.8)	
41–69	22,623	19728 (71.3)	2895 (10.5)	10251	9056 (71)	1195 (9.4)	
≥70	4007	3372 (12.2)	635 (2.3)	2072	1791 (14)	281 (2.2)	
Tumor size (6–50 mm) Mean ± SD	27,685 16.18 ± 7.7	23,937 (86.5)	3748 (13.5)	12,763 16.02 ± 7.7	11,185 (87.6)	1578 (12.4)	.044
T1b	6581	5972 (21.6)	609 (2.2)	3197	2948 (23.1)	249 (2.0)	
T1c	14,961	13105 (47.3)	1856 (6.7)	6772	6009 (47.1)	763 (6.0)	
T2	6143	4860 (17.6)	1283 (4.6)	2794	2228 (17.5)	566 (4.4)	
Histologic type	27,685	23937 (86.5)	3748 (13.5)	12,763	11185 (87.6)	1578 (12.4)	>.05
IDC	21,664	18235 (65.9)	3429 (12.4)	9931	8509 (66.7)	1422 (11.1)	
ILC	3023	2896 (10.5)	127 (0.5)	1466	1405 (11.0)	61 (0.5)	
IDC + ILC	1926	1831 (6.6)	95 (0.3)	897	853 (6.7)	44 (0.3)	
IDC + others	1072	975 (3.5)	97 (0.4)	469	418 (3.3)	51 (0.4)	
Histologic grade	27,685	23937 (86.5)	3748 (13.5)	12,763	11185 (87.6)	1578 (12.4)	.30
Grade 1	8516	8349 (30.2)	167 (0.6)	3955	3887 (30.5)	68 (0.5)	
Grade 2	14,906	13636 (49.3)	1270 (4.6)	6947	6438 (50.4)	509 (4.0)	
Grade 3	4263	1962 (7.1)	2311 (8.3)	1861	860 (6.7)	1001 (7.8)	
**LVI**	27685	23937 (86.5)	3748 (13.5)	12763	11185 (87.6)	1578 (12.4)	.98
Not present	24,885	21767 (78.6)	3118 (11.3)	11,471	10171 (79.7)	1300 (10.2)	
Present	2800	2170 (7.8)	630 (2.3)	1292	1014 (7.9)	278 (2.2)	
Progesterone receptor	27,685	23937 (86.5)	3748 (13.5)	12,763	11185 (87.6)	1578 (12.4)	.005
Positive	25,419	23128 (83.5)	2291 (8.3)	11,821	10821 (84.8)	1000 (7.8)	
Negative	2266	809 (2.9)	1457 (5.3)	942	364 (2.9)	578 (4.5)	

*SD* standard deviation, *IDC* invasive ductal carcinoma, *ILC* invasive lobular carcinoma, *IDC* *+* *ILC* invasive ductal and lobular carcinoma, *IDC* *+* *others* invasive ductal carcinoma mixed with other types, *LVI* lymph-vascular invasion

**Table 2:** Frequencies of a low-risk and a high risk OncotypeDX (ODX) recurrence score with TAILORx-trial cut-off values and pathologic descriptive characteristics

Characteristics	Total no	Original cohort NCDB 2010-2012 TAILORx-trial cut-off values		No	Validation cohort NCDB 2013 TAILORx-trial cut-off values		*p* value
		ODX low risk (0–10) No (% total)	ODX high risk (26-100) No (% total)		ODX low risk (0–10) No (% total)	ODX high risk (26-100) No (% total)	
Sex—Female	15,623	8919 (57.1)	6704 (42.9)	7454	4642 (62.3)	2812 (37.7)	
Age (21–90) Mean ± SD	15,623 59.32 ± 10.3	8919 (57.1)	6704 (42.9)	7454 59.61 ± 10.4	4642 (62.3)	2812 (37.7)	.043
≤40	558	222 (1.4)	336 (2.2)	285	118 (1.6)	167 (2.2)	
41–69	12,490	7180 (46.0)	5310 (34.0)	5879	3677 (49.3)	2202 (29.5)	
≥70	2575	1517 (9.7)	1058 (6.8)	1290	847 (11.4)	443 (5.9)	
Tumor size (6–50 mm) Mean ± SD	15,623 16.68 ± 7.9	8919 (57.1)	6704 (42.9)	7454 16.46 ± 8.0	4642 (62.3)	2812 (37.7)	.052
T1b	3424	2211 (14.2)	1213 (7.8)	1800	1292 (17.3)	508 (6.8)	
T1c	8313	4857 (31.1)	3456 (22.1)	3840	2434 (32.7)	1406 (18.9)	
T2	3886	1851 (11.8)	2035 (13.0)	1814	916 (12.3)	898 (12.0)	
Histologic type	15,623	8919 (57.1)	6704 (42.9)	7454	4642 (62.3)	2812 (37.7)	.98
IDC	12,810	6965 (44.6)	5845 (37.4)	6111	3670 (49.2)	2441 (32.7)	
ILC	1283	875 (5.6)	408 (2.6)	618	458 (6.1)	160 (2.1)	
IDC + ILC	920	646 (4.1)	274 (1.8)	441	320 (4.3)	121 (1.6)	
IDC + others	610	433 (2.8)	177 (1.1)	284	194 (2.6)	90 (1.2)	
Histologic grade	15,623	8919 (57.1)	6704 (42.9)	7454	4642 (62.3)	2812 (37.7)	.01
Grade 1	3854	3324 (21.3)	530 (3.4)	1914	1696 (22.8)	218 (2.9)	
Grade 2	7839	5016 (32.1)	2823 (18.1)	3797	2653 (35.6)	1144 (15.3)	
Grade 3	3930	579 (3.7)	3351 (21.4)	1743	293 (3.9)	1450 (19.5)	
LVI	15,623	8919 (57.1)	6704 (42.9)	7454	4642 (62.3)	2812 (37.7)	.34
Not present	13,698	8112 (51.9)	5586 (35.8)	6568	4262 (57.2)	2306 (30.9)	
Present	1925	807 (5.2)	1118 (7.2)	886	380 (5.1)	506 (6.8)	
Progesterone receptor	15,623	8919 (57.1)	6704 (42.9)	7454	4642 (62.3)	2812 (37.7)	.001
Positive	13,402	8790 (56.3)	4612 (29.5)	6575	4582 (61.5)	1993 (26.7)	
Negative	2221	129 (0.8)	2092 (13.4)	879	60 (0.8)	819 (11.0)	

*SD* standard deviation, *IDC* invasive ductal carcinoma, *ILC* invasive lobular carcinoma, *IDC* *+* *ILC* invasive ductal and lobular carcinoma, *IDC* *+* *others* invasive ductal carcinoma mixed with other types, *LVI* lymph-vascular invasion

In multivariate logistic regression analysis, age, tumor size, tumor grade, PR status, LVI, and histologic tumor type were significantly associated with a low-risk or a high-risk ODX recurrence score test result (Online Resource tables A1 through A4). The tumor grade and PR status showed the highest odds ratios: for example, grade 1 tumor was 49.42 times more likely to be associated with a low-risk recurrence score than a grade 3 tumor (95% CI 41.37–59.03, *p* < .001, commercial cut-off values); negative PR was 0.052 times less likely to be associated with a low-risk recurrence score (95% CI .046–.059, *p* < .001; Table A1). In Appendix online only Tables A1 through A4, the results of the final logistic regression analysis of six clinicopathologic variables used for creation of the nomograms are listed including *β* values.

Four nomograms were developed based on these analyses in the original cohort group (*n* = 27,685 for commercial ODX cut-off values; *n* = 15,623 for TAILORx-trial ODX cut-off values; points assigned for nomograms shown in Online Resource Tables A5 and A6. There was no evidence of multicollinearity found in our model. The overall predictive accuracy of the model (c-index) measured by the ROC curve was .887 (95% CI .880–.893) for commercial ODX cut-off recurrence score values and .851 (95% CI .845–.857) for TAILORx-trial cut-off values.

**Fig. 1:**
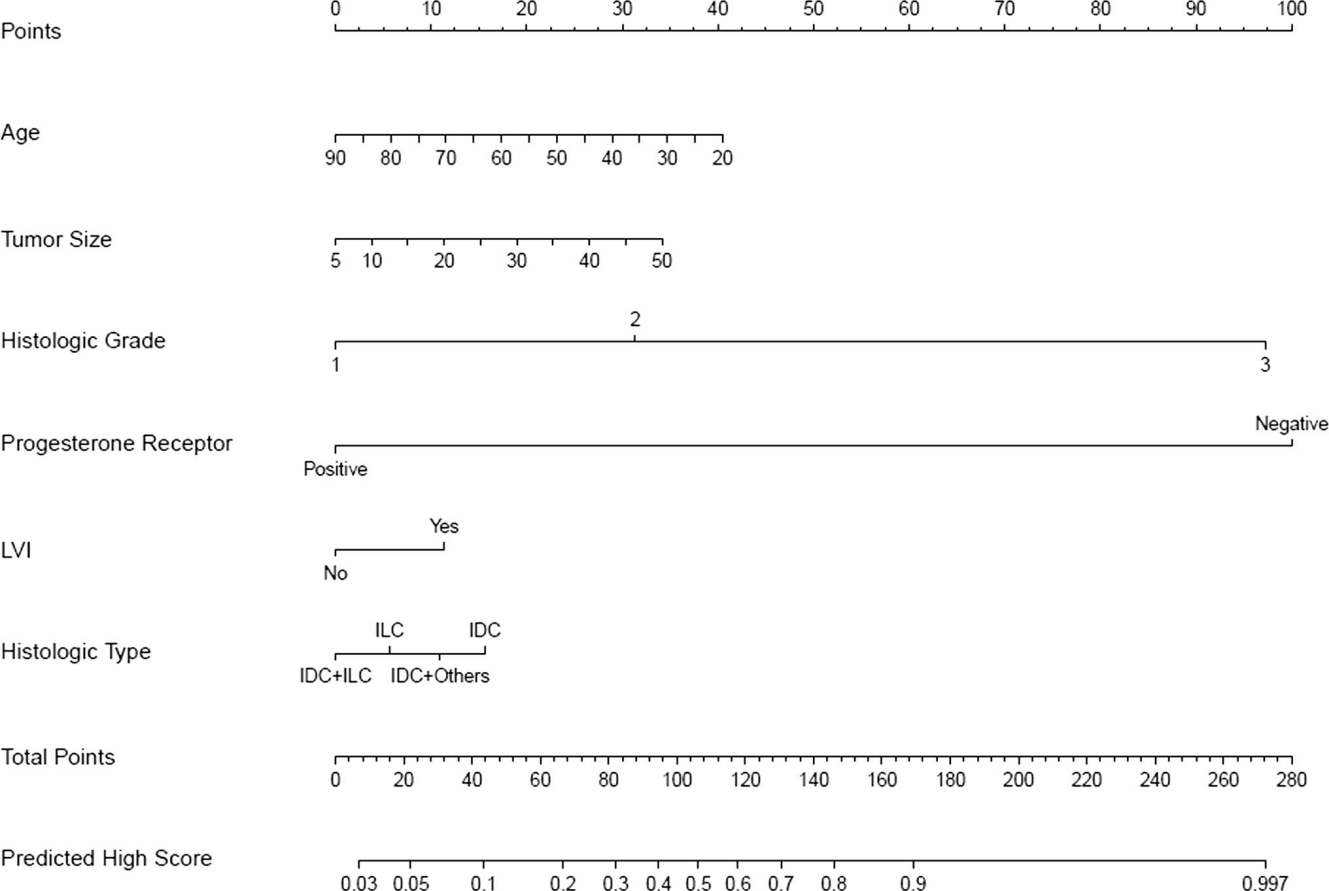
Nomogram to predict for high-risk Oncotype DX (ODX) score (TAILORx-trial cut-off values 26–100). *IDC* invasive ductal carcinoma, *ILC* invasive lobular carcinoma, *IDC* + *ILC* invasive ductal and lobular carcinoma, *IDC* + *others* invasive ductal carcinoma mixed with other types

**Fig. 2:**
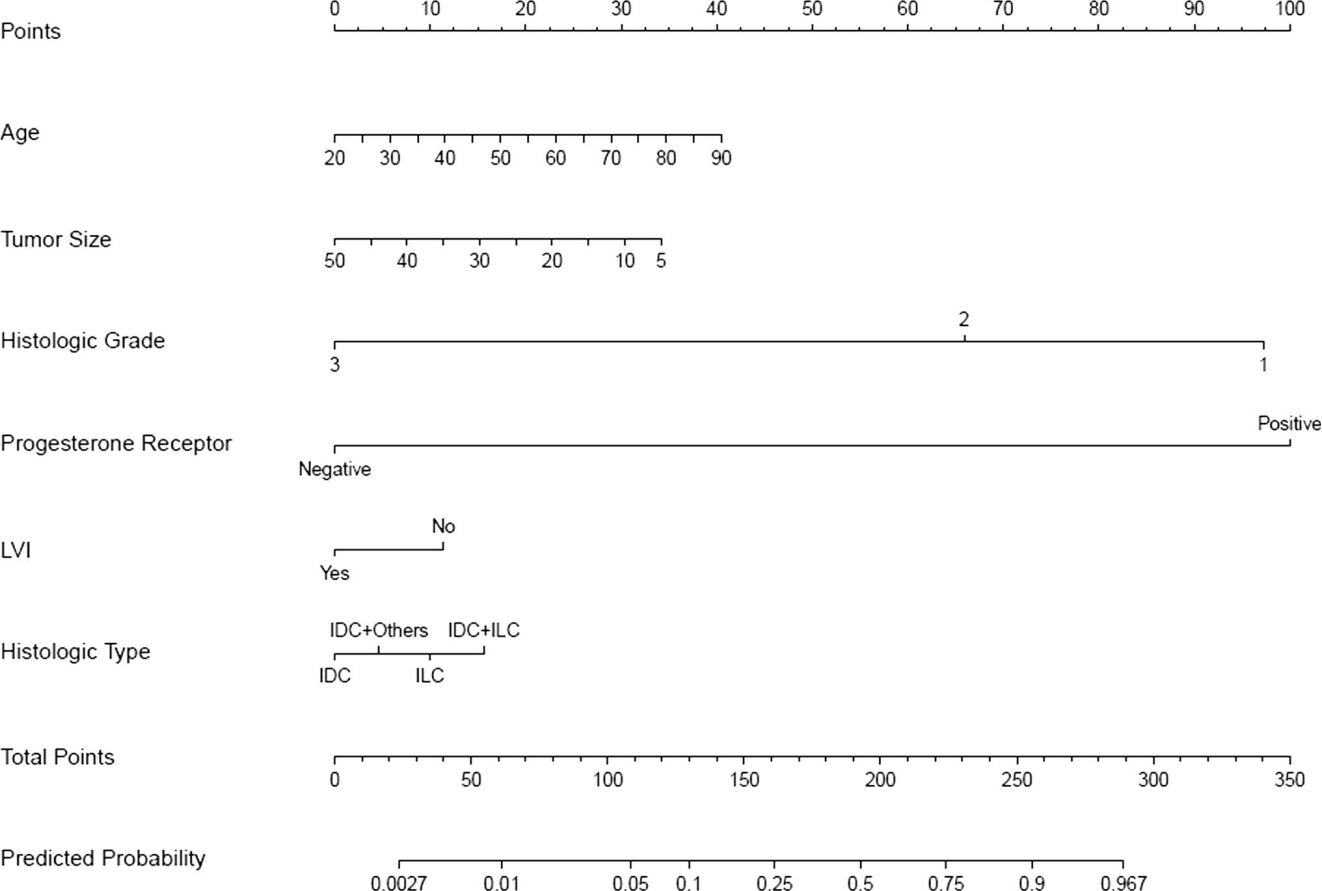
Nomogram to predict for a low-risk Oncotype DX (ODX) score (TAILORx-trial cut-off values 0–10). *IDC* invasive ductal carcinoma, *ILC* invasive lobular carcinoma, *IDC* + *ILC* invasive ductal and lobular carcinoma, *IDC + others* invasive ductal carcinoma mixed with other types

**Fig. 3:**
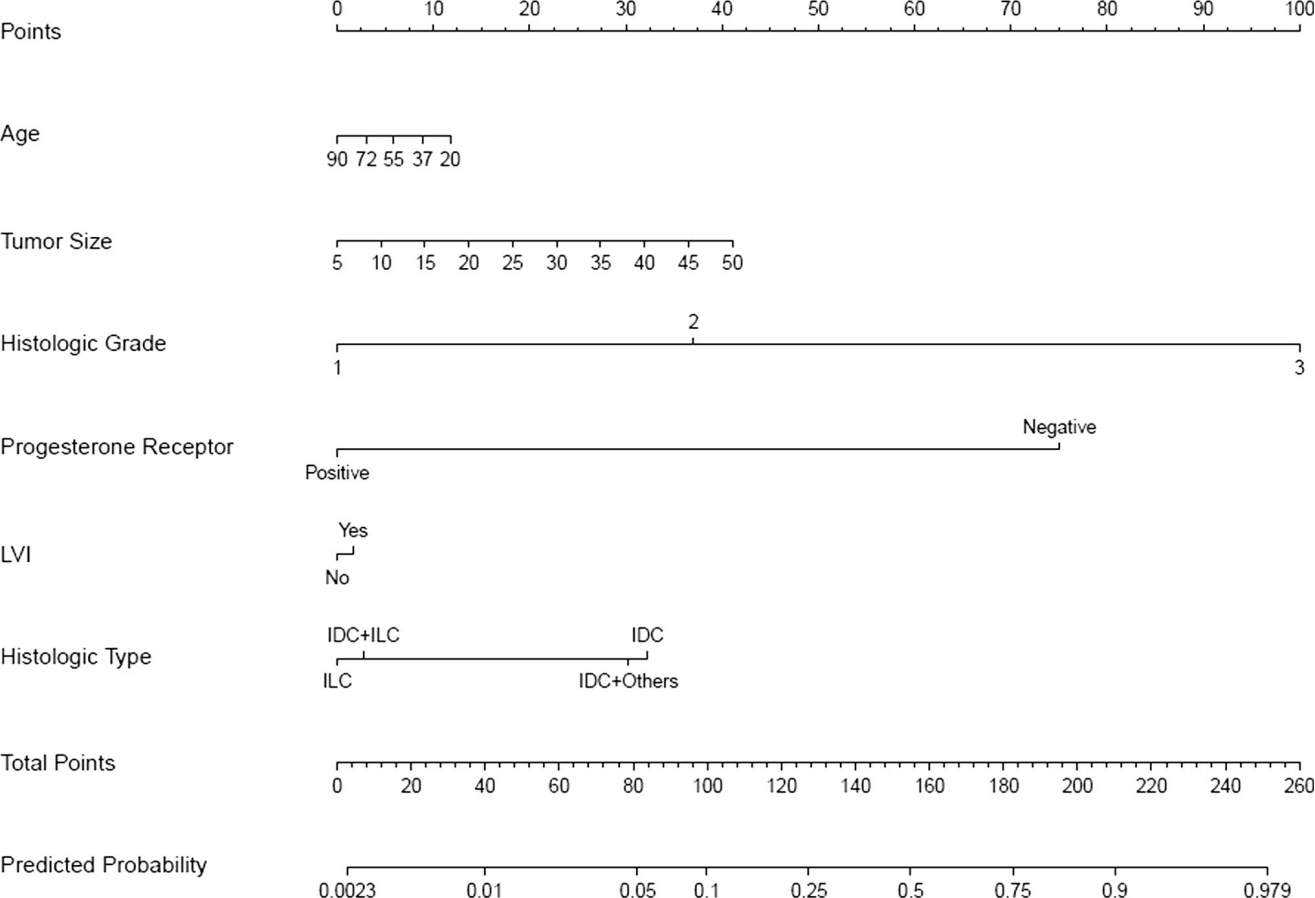
Nomogram to predict for high-risk Oncotype DX (ODX) score (commercial cut-off values 31–100). *IDC* invasive ductal carcinoma, *ILC* invasive lobular carcinoma, *IDC* + *ILC* invasive ductal and lobular carcinoma, *IDC + others* invasive ductal carcinoma mixed with other types

**Fig. 4:**
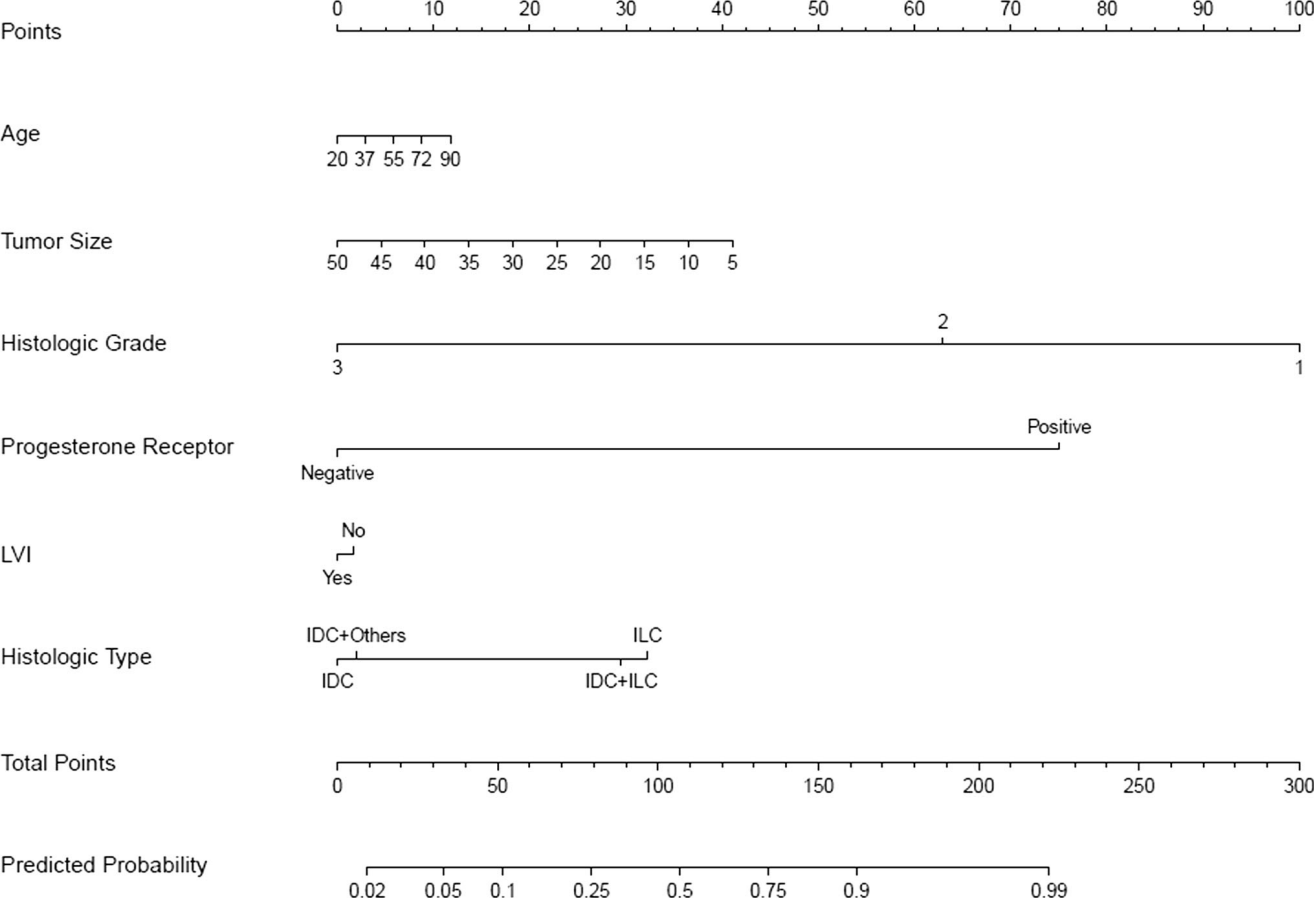
Nomogram to predict for low-risk Oncotype DX (ODX) score (commercial cut-off values 0–17). *IDC* invasive ductal carcinoma, *ILC* invasive lobular carcinoma, *IDC* + *ILC* invasive ductal and lobular carcinoma, *IDC + others* invasive ductal carcinoma mixed with other types

Four nomograms in the external validation group were developed (*n* = 12,685 for commercial ODX cut-off values; *n* = 7454 for TAILORx-trial ODX cut-off values) and are shown in Figs. 1, 2, 3, and 4; points assigned for nomograms are shown in Online Resource Tables A5 and A6. Source code is presented in Online Resource document A1. There was no evidence of multicollinearity. The overall predictive accuracy of the model (c-index) measured by the ROC curve was .89 (95% CI .88–.90) for commercial ODX cut-off values and .852 (95% CI .842–.861) for TAILORx-trial ODX cut-off values.

It was established that from six chosen clinicopathologic variables, tumor grade and PR status had the highest significant impact on predicting a high-risk or a low-risk ODX recurrence score with either commercial cut-off values or TAILORx-trial cut-off values, followed by age, histologic tumor type and tumor size, while LVI had minimal impact (Figs. 1, 2, 3 and 4 and Online Resource Tables A5 and A6).

### Utilizing the nomograms

Each nomogram (Figs. 1, 2, 3, and 4) consists of nine rows. For an individual patient, each of the six variables is assigned point values based on their clinicopathologic characteristics (topmost scale; see also Online Resource Tables A5 and A6 for assigned points). A vertical line is drawn between the variable value and the topmost “points” line. All allotted points for six variables are added, and the total is found in the “total points” row. A vertical line is then drawn between the final “total points” row and the “predicted probability” row, assigning the final predicted probability for a high-risk or a low-risk ODX recurrence score results for an individual patient.

Nomograms from the external validation cohort were used for development of our user-friendly online nomogram calculator in order to expedite the calculations of the probability of a low-risk or a high-risk ODX score for each patient (https://gsm.utmck.edu/nomograms).

## Discussion

ODX was the most commonly utilized breast cancer genomic assay in the United States recorded by the NCDB in the time period 2010–2012: from 202,075 ER+/HER2-negative/lymph node-negative breast cancer patients, 69,415 (34%) had genomic tests performed with 97% of tests being ODX. ODX is expensive (the current estimated cost is ~$4000 [22]), a factor which contributes to the test being performed in only approximately one-third of breast cancer patients in the USA [6] and in <20% of patients in European countries [7]. Several recent institution-based studies performed on a limited number of patients have used some of the pathologic variables routinely available from pathology reports in order to predict ODX test results [11–13, 15, 23–27]. Some of these studies have suggested that ordering ODX test in certain cases would not significantly contribute to clinical management decisions [11, 15, 27]. However, all of these studies were performed on a limited number of patients (institution based). A couple of studies used “H-score” for ER and PR scoring system [12, 27] which is more prone to interobserver variability. Kim et al. used a different low-risk and high-risk scoring system [15] in which a low-risk recurrence score incorporated TAILORx-trial low and intermediate recurrence scores (≤25), and a high score represented TAILORx-trial high-risk recurrence score (≥26) [28]. The majority of these studies also used Ki-67 proliferation index, in spite of the lack of consensus on scoring [16], which is also recognized by the newest 8^th^ edition of the American Joint Commission on Cancer (AJCC), which will be in use from January 2018 [29]. Ki-67 proliferation index has only “AJCC Level of Evidence: III” in the new AJCC edition.

We have developed novel, user-friendly nomograms as a surrogate prediction model for ODX test based on the large dataset from the NCDB of ODX-tested ER +/HER2-/lymph node-negative breast cancer patients. We used age, tumor size, tumor grade, PR status, LVI, and the four most common histologic types of breast cancer for predicting ODX test results. These six clinicopathologic variables were established in clinical practice as prognostic and/or predictive and are available from any pathology report. We found that tumor grade and PR status carried the highest predictive value for a low-risk or a high-risk ODX score, which is concordant with results reported by Gage et al. [11] in 540 patients gathered from three different institutions. Finding the grade as the highest predictor for ODX score is not surprising, since the grade was recognized as a significant predictor of breast cancer prognosis many years ago by the Nottingham Prognostic Index [30, 31] as well as the surrogate of proliferation at the St. Gallen Consensus Conference in 2011 [32]. In addition, high tumor grade and the 21-gene recurrence score were found as significant predictors of distant recurrence in the founding study of ODX test reported by Paik et al. [1].

PR was also a major predictor of a high-risk or a low-risk ODX recurrence score in our study, confirming recent observation by Chaudhary et al. in which PR-negative status was associated with higher ODX scores [14]. Our study is the first to show the predictive value of histologic tumor type, age, and tumor size to predict ODX test results. Older patients were less likely to have a high-risk score; larger tumors were more likely to have a high-risk score in our study. These observations were not described in the original study of 21-multigene assay [1], or in a recently published study on optimizing the use of gene expression profiling by Kim et al. [15] perhaps due to the relatively small number of tested patients in those studies in comparison to number of patients in our study. LVI had minimal impact on prediction of ODX test results in our model.

Our nomograms can be used for predicting ODX test results with both TAILORx-trial and commercial cut-off score models. These nomograms therefore accommodate for the differences of ODX scoring results currently in use and allow the interchangeable use of our nomograms/calculators to the clinician’s preferences. We believe that these options give our study advantage over other studies which did not use both commercial and TAILORx-trial cut-off values [11, 12, 15].

ER and PR positivity in our study was established based on the ASCO/CAP guidelines as ≥1% of positive staining cells [33] (guidelines followed by NCDB registrars). ER and PR status was recorded as either positive or negative in the NCDB without associated levels of positivity. While this could be considered as a weakness of our study, we believe that it was actually a strength, since it confirmed the robustness of our prediction model even without use of additional information such as level of positive expression or the intensity of immunohistochemical staining of ER and PR. Another strength of our study is supported by the large datasets from the National Cancer Data Base. Large number of patients in both the original and the external validation cohorts (27,685 and 12,763, respectively) were used to develop nomograms which had high, acceptable C-indexes (.85–.89). This is the first study of this scale showing confidently that clinicopathologic variables can be used for prediction of low-risk or high-risk ODXRS using our nomogram models.

In the 8th edition of the American Joint Commission on Cancer (AJCC), which will be in use beginning of January 2018, hormone receptor-positive/HER2-negative and lymph node-negative breast cancer patients with a low-risk recurrence score of multigene breast cancer prognostic panels, such as ODX, Mammaprint, EndoPredict, PAM 50, and Breast Cancer Index, have been placed into the same prognostic category as T1a-T1b N0 M0 tumors, regardless of T size. This intent together with the newest 2016 ASCO clinical practice guidelines for use of biomarkers to guide clinical decisions regarding the use of adjuvant systemic therapy for women with early-stage invasive breast carcinoma has embraced the use of genomic prognostic assays as an ideal way of practicing personalized medicine for each breast cancer patient. Unfortunately, these tests are expensive and are not affordable or available for the majority of the breast cancer patients globally. Therefore, our nomograms which predict for a high-risk or a low-risk ODX recurrence score will be useful tools to assist providers in selecting patients for which further ODX testing may or may not be necessary. They also may serve as a surrogate for patients for which ODX testing is not affordable or available.

## Electronic supplementary material

Below is the link to the electronic supplementary material.

Supplementary material 1 (DOCX 38 kb)
