# Supplementary material for: Oncotype DX breast cancer recurrence score can be predicted with a novel nomogram using clinicopathologic data
Source: Breast Cancer Res Treat. 2017 Feb 27;163(1):51–61. doi: 10.1007/s10549-017-4170-3 (PMC5387031; doi:10.1007/s10549-017-4170-3)
Supplement: Supplementary file 1 — Supplementary material 1 (DOCX 38 kb) [file 10549_2017_4170_MOESM1_ESM.docx]

**ONLINE RESOURCE**

**Title:** Oncotype DX breast cancer recurrence score can be predicted with a novel nomogram using clinicopathologic data

**Authors:** Amila Orucevic, MD, PhD^1*^, John L. Bell, MD^2^, Alison P. McNabb, MSc^3^ and Robert E. Heidel, PhD^2^

**Authors affiliations:**

^1*^Departments of Pathology, University of Tennessee Medical Center, 1924 Alcoa Hwy, Knoxville, Tennessee, 37920, USA

^2^Department of Surgery, University of Tennessee Medical Center, 1924 Alcoa Hwy, Knoxville, Tennessee, 37920, USA

^3^Graduate School of Medicine, University of Tennessee Medical Center, 1924 Alcoa Hwy, Knoxville, Tennessee, 37920, USA

***Corresponding author**: Amila Orucevic, MD, PhD, Department of Pathology, University of Tennessee Medical Center at Knoxville, Knoxville, TN, 37920,

Phone: 865-305-9080, Fax: 865-305-6866, e-mail: [aorucevic@utmck.edu](mailto:aorucevic@utmck.edu)

**Online resource Table A1.** Logistic regression analysis of original and external validations cohorts for predicting a low-risk Oncotype DX recurrence score test results with commercial cut-off values for a low-risk score (0-17)

|  | **Original cohort (N=27685)** | | | | | **External validation cohort (N=12763)** | | | | |
| --- | --- | --- | --- | --- | --- | --- | --- | --- | --- | --- |
|  | **β** | ***p*** | **CI** | **95% CI** | | **β** | ***p*** | **CI** | **95% CI** | |
|  |  |  |  | **Lower** | **Upper** |  |  |  | **Upper** | **Lower** |
| **Age** | .005 | .014 | 1.005 | 1.001 | 1.01 | .02 | <.001 | 1.02 | 1.014 | 1.026 |
| **Tumor size** | -.032 | <.001 | .968 | .963 | .974 | -.027 | <.001 | .974 | .966 | .981 |
| **Grade 3** | Referent <.001 | | | | | Referent <.001 | | | | |
| **Grade 2** | 2.35 | <.001 | 10.48 | 9.53 | 11.54 | 2.305 | <.001 | 10.02 | 8.59 | 11.69 |
| **Grade 1** | 3.9 | <.001 | 49.42 | 41.37 | 59.03 | 3.401 | <.001 | 30.0 | 24.42 | 36.84 |
| **PR positive** | Referent <.001 | | | | | Referent <.001 | | | | |
| **PR negative** | -2.962 | <.001 | .052 | .046 | .059 | -3.498 | <.001 | .030 | .023 | .040 |
| **LVI not present** | Referent <.001 | | | | | Referent <.001 | | | | |
| **LVI present** | -.025 | .702 | .976 | .860 | 1.107 | -.399 | <.001 | .671 | .559 | .807 |
| **IDC** | Referent <.001 | | | | | Referent <.001 | | | | |
| **ILC** | 1.407 | <.001 | 4.083 | 3.314 | 5.03 | .348 | .003 | 1.416 | 1.125 | 1.784 |
| **IDC+ILC** | 1.223 | <.001 | 3.397 | 2.685 | 4.298 | .546 | <.001 | 1.726 | 1.315 | 2.265 |
| **IDC + others** | .294 | .030 | 1.342 | 1.028 | 1.751 | .162 | .335 | 1.176 | .846 | 1.635 |

Table legend: PR – progesterone receptor; IDC – invasive ductal carcinoma; ILC – invasive lobular carcinoma; IDC + ILC – invasive ductal and lobular carcinoma; IDC + others – invasive ductal carcinoma mixed with other types; LVI – lymph-vascular invasion

**Online resource Table A2.** Logistic regression analysis of original and external validation cohorts for predicting a high-risk Oncotype DX recurrence score test results with commercial cut-off values for a high-risk score (31-100)

|  | **Original cohort (N=27685)** | | | | | **External validation cohort (N=12763)** | | | | |
| --- | --- | --- | --- | --- | --- | --- | --- | --- | --- | --- |
|  | **β** | ***p*** | **CI** | **95% CI** | | **β** | ***p*** | **CI** | **95% CI** | |
|  |  |  |  | **Lower** | **Upper** |  |  |  | **Upper** | **Lower** |
| **Age** | -.005 | .014 | .995 | .991 | .999 | -.007 | .042 | .993 | .987 | 1.000 |
| **Tumor size** | .032 | <.001 | 1.033 | 1.027 | 1.039 | .036 | <.001 | 1.037 | 1.029 | 1.046 |
| **Grade 3** | Referent <.001 | | | | | Referent <.001 | | | | |
| **Grade 2** | -2.35 | <.001 | .095 | .087 | .105 | -2.512 | <.001 | .081 | .070 | .094 |
| **Grade 1** | -3.9 | <.001 | .020 | .017 | .024 | -3.99 | <.001 | .019 | .014 | .024 |
| **PR positive** | Referent <.001 | | | | | Referent <.001 | | | | |
| **PR negative** | 2.962 | <.001 | 19.33 | 17.06 | 21.906 | 2.995 | <.001 | 19.976 | 16.429 | 24.289 |
| **LVI not present** | Referent <.001 | | | | | Referent <.001 | | | | |
| **LVI present** | .025 | .702 | 1.025 | .904 | 1.162 | .067 | .493 | 1.069 | .884 | 1.293 |
| **IDC** | Referent <.001 | | | | | Referent <.001 | | | | |
| **ILC** | -1.407 | <.001 | .245 | .199 | .302 | -1.286 | <.001 | .276 | .205 | .373 |
| **IDC+ILC** | -1.223 | <.001 | .294 | .233 | .372 | -1.177 | <.001 | .308 | .217 | .438 |
| **IDC + others** | -.294 | .030 | .745 | .571 | .972 | -.078 | .689 | .925 | .630 | 1.357 |

Table legend: PR – progesterone receptor; IDC – invasive ductal carcinoma; ILC – invasive lobular carcinoma; IDC + ILC – invasive ductal and lobular carcinoma; IDC + others – invasive ductal carcinoma mixed with other types; LVI – lymph-vascular invasion

**Online resource Table A3.** Logistic regression analysis of original and external validation cohorts for predicting a low-risk Oncotype DX recurrence score test results with TAILORx trial cut-off values for a low-risk score (0-10)

|  | **Original cohort (N=27685)** | | | | | **External validation cohort (N=12763)** | | | | |
| --- | --- | --- | --- | --- | --- | --- | --- | --- | --- | --- |
|  | **β** | ***p*** | **CI** | **95% CI** | | **β** | ***p*** | **CI** | **95% CI** | |
|  |  |  |  | **Lower** | **Upper** |  |  |  | **Upper** | **Lower** |
| **Age** | .018 | <.001 | 1.019 | 1.015 | 1.023 | .02 | <.001 | 1.020 | 1.014 | 1.026 |
| **Tumor size** | -.016 | <.001 | .984 | .979 | .990 | -.027 | <.001 | .974 | .966 | .981 |
| **Grade 3** | Referent <.001 | | | | | Referent <.001 | | | | |
| **Grade 2** | 2.20 | <.001 | 9.024 | 8.113 | 10.036 | 2.305 | <.001 | 10.026 | 8.597 | 11.692 |
| **Grade 1** | 3.465 | <.001 | 31.992 | 27.786 | 36.834 | 3.401 | <.001 | 30.001 | 24.427 | 36.846 |
| **PR positive** | Referent <.001 | | | | | Referent <.001 | | | | |
| **PR negative** | -3.484 | <.001 | .031 | .025 | .037 | -3.498 | <.001 | .030 | .023 | .040 |
| **LVI not present** | Referent <.001 | | | | | Referent <.001 | | | | |
| **LVI present** | -.190 | .003 | .827 | .730 | .937 | -.399 | <.001 | .671 | .559 | .807 |
| **IDC** | Referent <.001 | | | | | Referent <.001 | | | | |
| **ILC** | .355 | <.001 | 1.426 | 1.223 | 1.663 | .348 | .003 | 1.416 | 1.125 | 1.784 |
| **IDC+ILC** | .531 | <.001 | 1.701 | 1.424 | 2.033 | .546 | <.001 | 1.726 | 1.315 | 2.265 |
| **IDC + others** | .484 | <.001 | 1.622 | 1.288 | 2.043 | .162 | .335 | 1.176 | .846 | 1.635 |

Table legend: PR – progesterone receptor; IDC – invasive ductal carcinoma; ILC – invasive lobular carcinoma; IDC + ILC – invasive ductal and lobular carcinoma; IDC + others – invasive ductal carcinoma mixed with other types; LVI – lymph-vascular invasion

**Online resource Table A4.** Logistic regression analysis of original and external validation cohorts for predicting a high-risk Oncotype DX recurrence score test results with TAILORx trial cut-off values for a high-risk score (26-100)

|  | **Original cohort (N=27685)** | | | | | **External validation cohort (N=12763)** | | | | |
| --- | --- | --- | --- | --- | --- | --- | --- | --- | --- | --- |
|  | **β** | ***p*** | **CI** | **95% CI** | | **β** | ***p*** | **CI** | **95% CI** | |
|  |  |  |  | **Lower** | **Upper** |  |  |  | **Upper** | **Lower** |
| **Age** | -.018 | <.001 | .982 | .978 | .986 | -.02 | <.001 | .980 | .974 | .986 |
| **Tumor size** | .016 | <.001 | 1.016 | 1.011 | 1.021 | .027 | <.001 | 1.027 | 1.019 | 1.035 |
| **Grade 3** | Referent <.001 | | | | | Referent <.001 | | | | |
| **Grade 2** | -2.20 | <.001 | .111 | .10 | .123 | -2.305 | <.001 | .10 | .086 | .116 |
| **Grade 1** | -3.465 | <.001 | .031 | .027 | .036 | -3.401 | <.001 | .033 | .027 | .041 |
| **PR positive** | Referent <.001 | | | | | Referent <.001 | | | | |
| **PR negative** | 3.484 | <.001 | 32.58 | 26.83 | 39.56 | 3.498 | <.001 | 33.04 | 24.79 | 44.02 |
| **LVI not present** | Referent <.001 | | | | | Referent <.001 | | | | |
| **LVI present** | .190 | .003 | 1.209 | 1.068 | 1.369 | .399 | <.001 | 1.49 | 1.24 | 1.79 |
| **IDC** | Referent <.001 | | | | | Referent <.001 | | | | |
| **ILC** | -.355 | <.001 | .701 | .601 | .818 | -.348 | .003 | .706 | .560 | .889 |
| **IDC+ILC** | -.531 | <.001 | .588 | .492 | .702 | -.546 | <.001 | .579 | .441 | .761 |
| **IDC + others** | -.484 | <.001 | .616 | .490 | .776 | -.162 | .335 | .85 | .612 | 1.182 |

Table legend: PR – progesterone receptor; IDC – invasive ductal carcinoma; ILC – invasive lobular carcinoma; IDC + ILC – invasive ductal and lobular carcinoma; IDC + others – invasive ductal carcinoma mixed with other types; LVI – lymph-vascular invasion

**Online resource Table A5.** Points assigned for a probability of a high-risk or a low-risk Oncotype DX (ODX) recurrence score (RS) test results in original and external validation cohort nomograms with commercial high-risk and low-risk cut-off values

|  | | ***Points assigned for original cohort nomogram (2010-2012; N=27,685)***  ***Commercial cut-off values*** | | ***Points assigned for external validation cohort nomogram (2013; N=12,763)***  ***Commercial cut-off values*** | |
| --- | --- | --- | --- | --- | --- |
| **Clinicopathologic Characteristics** | | **High-risk ODX RS**  **(31-100)** | **Low-risk ODX RS**  **(0-17)** | **High-risk ODX RS**  **(31-100)** | **Low-risk ODX RS**  **(0-17)** |
| **Age (20-90)** | | 8.9 | | 11.8 | |
| **Tumor size**  **(6-50 mm)** | | 36.1 | | 40.14 | |
| **Grade** | **1** | 0 | 100 | 0 | 100 |
|  | **2** | 40 | 60 | 37.1 | 62.9 |
|  | **3** | 100 | 0 | 100 | 0 |
| **PR** | **Positive ≥1%** | 0 | 75.9 | 0 | 75 |
|  | **Negative <1%** | 75.9 | 0 | 75 | 0 |
| **LVI** | **Yes** | 0.6 | 0 | 1.6 | 0 |
|  | **No** | 0 | 0.6 | 0 | 1.6 |
| **Histologic type of invasive BC** | **IDC** | 36 | 0 | 32.2 | 0 |
|  | **ILC** | 0 | 36 | 0 | 32.2 |
|  | **IDC + ILC** | 7.5 | 31 | 1.9 | 29.4 |
|  | **IDC + others** | 31 | 7.5 | 29.4 | 1.9 |
| **Maximum points** | | 257.5 | | 260.7 | |
| **Probability** | | 0.003 per point | | 0.003376 per point | |
| **C-index (95% CI)** | | .887 (.880-.893) | | .89 (.88-.90) | |
| **P value** | | <.001 | | <.001 | |

Table legend: ODX – Oncotype DX; RS – recurrence score; PR – progesterone receptor; IDC – invasive ductal carcinoma; ILC – invasive lobular carcinoma; IDC + ILC – invasive ductal and lobular carcinoma; IDC + others – invasive ductal carcinoma mixed with other types; LVI – lymph-vascular invasion

**Online resource Table A6.** Points assigned for a probability of a high-risk or a low-risk Oncotype DX (ODX) recurrence score (RS) test results in original and external validation cohort nomograms with TAILORx trial high-risk and low-risk cut-off values

|  | | ***Points assigned for original cohort nomogram (2010-2012; N=27,685)***  ***TAILORx trial cut-off values*** | | ***Points assigned for external validation cohort nomogram (2013; N=12,763)***  ***TAILORx trial cut-off values*** | |
| --- | --- | --- | --- | --- | --- |
| **Clinicopathologic Characteristics** | | **High-risk ODX RS**  **(26-100)** | **Low-risk ODX RS**  **(0-10)** | **High-risk ODX RS**  **(26-100)** | **Low-risk ODX RS**  **(0-10)** |
| **Age (21-90)** | | 35.6 | | 39.9 | |
| **Tumor size**  **(6-50 mm)** | | 20.2 | | 33.3 | |
| **Grade** | **1** | 0 | 99.4 | 0 | 97.2 |
|  | **2** | 36.4 | 63 | 31.4 | 65.8 |
|  | **3** | 99.4 | 0 | 97.2 | 0 |
| **PR** | **Positive ≥1%** | 0 | 100 | 0 | 100 |
|  | **Negative <1%** | 100 | 0 | 100 | 0 |
| **LVI** | **Yes** | 5.4 | 0 | 11.4 | 0 |
|  | **No** | 0 | 5.4 | 0 | 11.4 |
| **Histologic type of invasive BC** | **IDC** | 15 | 0 | 15.6 | 0 |
|  | **ILC** | 13.8 | 10 | 4.6 | 9.9 |
|  | **IDC + ILC** | 0 | 15 | 0 | 15.6 |
|  | **IDC + others** | 10 | 13.8 | 9.9 | 4.6 |
| **Maximum points** | | 275.6 | | 297.4 | |
| **Probability** | | 0.003 per point | | 0.00325 per point | 0.003229 per point |
| **C-index (95% CI)** | | .851 (.845-.857) | | .852 (.842-.861) | |
| **P value** | | <.001 | | <.001 | |

Table legend: ODX – Oncotype DX; RS – recurrence score; PR – progesterone receptor; IDC – invasive ductal carcinoma; ILC – invasive lobular carcinoma; IDC + ILC – invasive ductal and lobular carcinoma; IDC + others – invasive ductal carcinoma mixed with other types; LVI – lymph-vascular invasion

**Online resource Document A1:**

Source code in R:

d<-read.csv("data.csv", header = TRUE, stringsAsFactors = TRUE)

attach(d)

library(rms)

dd<-datadist(Age, Size, Grade, CSF2, LVI, Histology, Outcome); options(datadist="dd")

fit<-lrm(Outcome~Age+Size+Grade+CSF2+LVI+Histology, x=TRUE, y=TRUE)

n<-nomogram(fit, fun=plogis, lp=FALSE, funlabel="Predicted Probability")

plot(n)
